# Nanoparticles for Thrombus Diagnosis and Therapy: Emerging Trends in Thrombus-theranostics

**DOI:** 10.7150/ntno.92184

**Published:** 2024-01-01

**Authors:** Samridhi, Aseem Setia, Abhishesh Kumar Mehata, Vishnu Priya, Aditi Pradhan, Pragya Prasanna, Syam Mohan, Madaswamy S Muthu

**Affiliations:** 1 Department of Pharmaceutical Engineering and Technology, Indian Institute of Technology (BHU), Varanasi-221005, India.; 2 National Institute of Pharmaceutical Education and Research, Hajipur, Bihar, India.; 3 Substance Abuse and Toxicology Research Centre, Jazan University, Jazan 45142, Saudi Arabia.; 4 School of Health Sciences, University of Petroleum and Energy Studies, Dehradun 248007, India.

**Keywords:** thrombosis, pathophysiology, animal models, nanoparticles, imaging

## Abstract

Cardiovascular disease is one of the chief factors that cause ischemic stroke, myocardial infarction, and venous thromboembolism. The elements that speed up thrombosis include nutritional consumption, physical activity, and oxidative stress. Even though the precise etiology and pathophysiology remain difficult topics that primarily rely on traditional medicine. The diagnosis and management of thrombosis are being developed using discrete non-invasive and non-surgical approaches. One of the emerging promising approach is ultrasound and photoacoustic imaging. The advancement of nanomedicines offers concentrated therapy and diagnosis, imparting efficacy and fewer side effects which is more significant than conventional medicine. This study addresses the potential of nanomedicines as theranostic agents for the treatment of thrombosis. In this article, we describe the factors that lead to thrombosis and its consequences, as well as summarize the findings of studies on thrombus formation in preclinical and clinical models and also provide insights on nanoparticles for thrombus imaging and therapy.

## 1. Introduction

Cardiovascular diseases (CVDs) account for an estimated 1 in every 4 deaths globally 1. In 2019, CVDs are expected to account for an estimated 32 percent, or 17.9 million, deaths worldwide. Heart disease and stroke accounted for 85% of these fatalities 2. In low and middle-income countries, CVDs is the leading cause of mortality. In 2019, CVDs accounted for 38 percent of the 17 million premature deaths (before the age of 70) caused by non-communicable diseases 3. Thrombotic disorders pose a substantial threat to human health cause ischemic stroke, myocardial infarction, and pulmonary thromboembolism, and are responsible for significant morbidity and death 4. One of the major causes of mortality in 2010 and one that causes one to four fatalities globally is thrombosis. Vein and arterial thrombosis are two types of thrombosis. The two most common arterial thrombosis are ischemic heart disease and stroke, whereas venous thromboembolism is made up of deep vein thrombosis, pulmonary thromboembolism, and other conditions 5.

All of the therapeutic/pharmacological agents that are available for the treatment and management of thrombosis have been in use for decades or older variations and replaced by newer products that only offer a tiny incremental improvement, despite tremendous advances in understanding the biology of thrombus formation and the pathophysiology of thrombosis. The multistep process of thrombosis occurs when endogenous anticoagulants and hemostasis are disturbed by a complicated pathological event 6. The three conventional risk factors for thrombosis are endothelium lining of the vascular wall, a hypercoagulable condition, and arterial or venous blood stasis 7. Endothelial damage, hypercoagulability, and arterial or venous blood stasis are together known as Virchow's triad. The virchow's triad is the major contributor to the etiology of thrombosis. When the artery wall is damaged, prothrombotic (and pro-inflammatory) cytokines are produced, tissue factor levels slightly change, adhesion molecules multiply, and platelet activation is increased 8. Leukocytes and endothelial cells interact to promote inflammation when cytokines are present. Inflammation is a typical bodily response to unfavorable stimuli, such as infection from external pathogens or endothelium damage, whether it is acute or chronic. Leucocyte and endothelial cell activation results in the production of adhesion molecules, which eventually lead to the development of clots 9. Through a complicated regulatory process that maintains homeostasis through the natural anticoagulants produced by the body, like protein C, S and AT-III (Antithrombin-III), which thwart the onset of the thrombosis 10.

The antithrombotic agents that are used to treat thrombosis are divided into three classes i.e., antiplatelets, anticoagulant, and thrombolytic agents. There are various side effects associated with the use of these antithrombotic agents like they can affect the normal coagulation function, and irrational use may cause bleeding complications. The constraints of traditional drug formulation properties have been overcome by nanoparticle-based drug delivery systems 11. A potent collection of nano-engineered devices is combined in nanomedicine, a branch of nanotechnology used for medical diagnostic and/or therapeutic purposes. The drug delivery which is based on nanoparticles (NPs) can lengthen the period of medication circulates in the body, boost therapeutic effectiveness, and lessen undesirable off-target effects 12, 13.

Current advancements in nanotechnology and nanoscience provide numerous opportunities in the diagnosis and treatment of several diseases such as Cardiovascular system (CVS), pulmonary and hematological, etc. The application of nanotechnologies in medicine offers enormous potential for enhanced biosensors and implants, targeted drug delivery, and tissue engineering 14. More specifically, the NPs have a high surface area to volume ratio in the size range of 10-100 nanometers, which permits the conjugation of several therapeutic and diagnostic substances (imaging/contrast agents) and their numerous interactions with cell membrane receptors, peptides, among other things. Due to their very small size, they may travel via blood arteries *in vivo* and deliver therapeutic or diagnostic substances to the target 15.

As theranostic NPs have several advantages: (1) multiple capabilities, including reduced immunogenicity, targeting, multimodal imaging, treatment, and controlled pharmacokinetics, are made possible by their modular structure and surface changes, (2) specific tissues can be passively targeted, (3) NPs can respond to the microenvironment 16, 17. These characteristics make NPs the best imaging agents available for use in conventional medical imaging, and they also make it possible to create novel modalities and theranostic applications 18. The readily available biological imaging NPs utilize a range of materials. They act as contrast agents in medical imaging, detectable concurrently with several modalities, and they inspire the development of new methods for the ever-richer gathering of molecular data 19. Since they lessen systemic adverse effects, several are already used in clinical settings as treatments or pharmaceutical delivery systems 20 21. Molecular imaging techniques have a great ability as less intrusive methods for the detection and treatment of numerous diseases. This imaging technique uses a combination of targeting and imaging moiety for disease-specific sites in the human. This emerging field links conventional medical imaging methods to envisage the operation of various biological processes *in vivo*. The main merit of this type of molecular imaging technique is the measurement of treatment response at very early time points and the ability to mark a medicine with an imaging agent to monitor a drug's bio-distribution 22. The latter is associated with theranostic, a relatively recent area that combines therapy with diagnostics to give targeted treatment in a regulated fashion and monitor response using molecular imaging at the same time 23. In a study, Vazquez-Prada *et al,* developed silver iron oxide NPs (AgIONPs) for photothermal therapy. The AgIONPs are designed to target thrombi by biofunctionalizing them with binding ligands. Once functionalized with a single chain antibody targeting active platelets, photoacoustic and fluorescence imaging showed that AgIONPs bind very specifically to the thrombus. While the non-targeted group does not experience a complete restoration of blood flow after photothermal thrombolysis in vivo, the targeted group does see an increase in thrombi temperature. Comparing the targeted groups' thrombolysis to the standard thrombolytic used in the clinic, there is a considerable improvement (p < 0.0001). There are no noticeable negative effects of AgIONPs in the assays. Taken together, the results of this study point to AgIONPs as a possible theranostic drug for thrombosis 24.

There is various imaging technique employed for the theranostic approach of nano-structured formulation and some are already under clinical practice at present time and aid in clinical decision-making, while others are presently at advanced development stages. The techniques used are Magnetic Resonance Imaging (MRI), Optical, Computer Tomography (CT), Ultra Sound (US), Photo Acoustic Imaging (PAI) 25, Positron Emission Tomography (PET), Single-Photon Emission Computer Tomography (SPECT), Near-infrared fluorescence (NIRF) etc. 26. Most thrombotic patients have essentially no noticeable symptoms in the early stages of thrombogenesis, which makes it far more difficult to diagnose and treat thrombus effectively 27. It is generally accepted that accurate early diagnosis is a prerequisite for timely avoidance of life-threatening thromboembolic events 28 29.

Thrombosis research relies on intricate *in vitro* models, involving simulated environments and cell cultures, to study cellular interactions and coagulation pathways*. In vivo* models, utilizing animal models like murine and primate models, closely mimic human thrombotic conditions 30. In this review, we summarize in detail about pathophysiology, animal modelling and various synthetic nanoprticles that are employed for *in vivo* and *in vitro* molecular imaging and therapy of thrombosis.

## 2. Pathophysiology and major steps in the development of a thrombus

**Figure 1:**
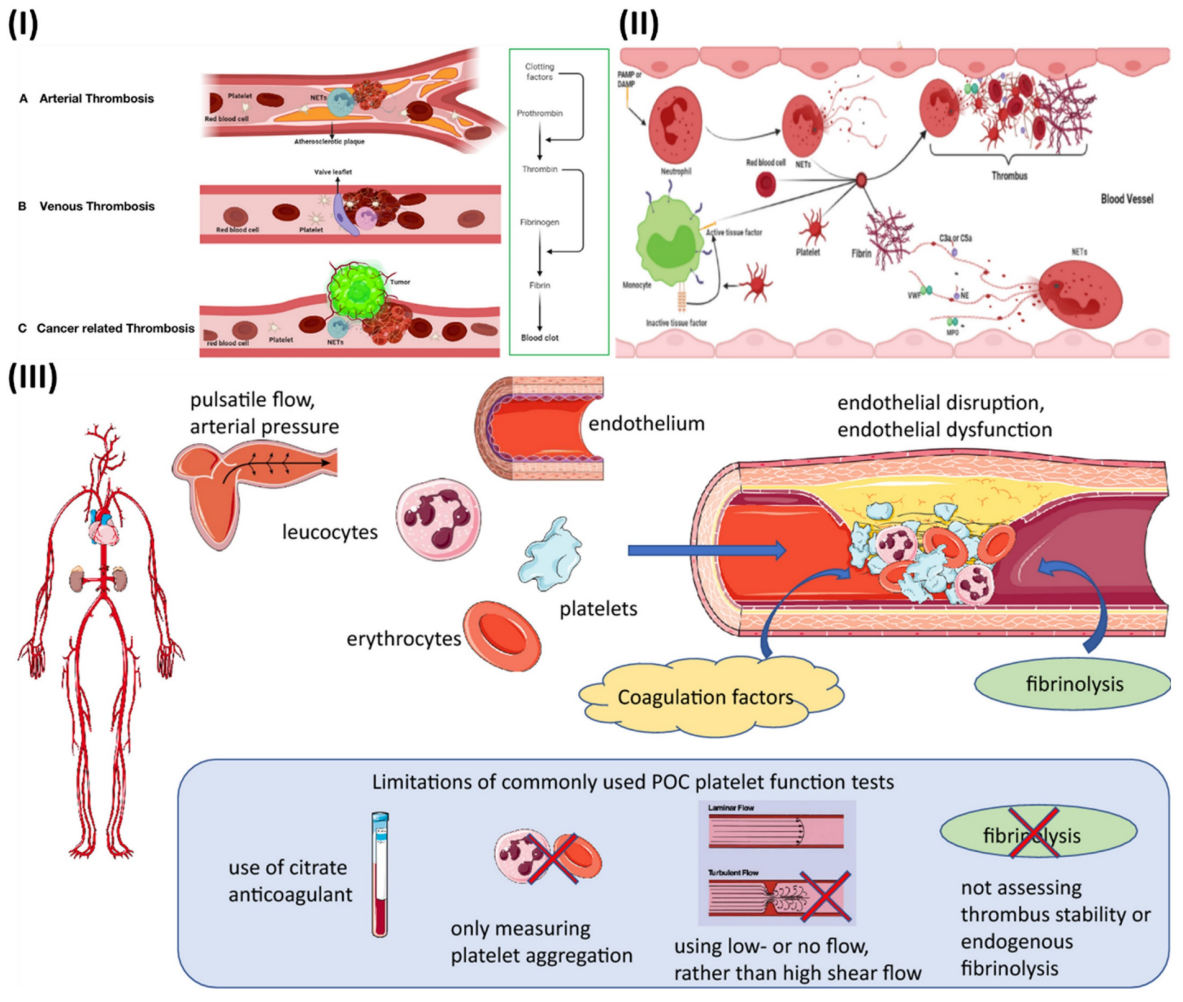
(I) Mechanisms of thrombus formation of arterial thrombosis (A), venous thrombosis (B), and cancer-related thrombosis (C); (II) Interaction between the various blood components and NETs in the process of thrombosis progression. Reproduce with the permission from ref 32. Fig. 2 and Fig. 3 (Frontiers); (III) The fundamental pathophysiological factors that contribute to arterial thrombosis and the limits of point-of-care (POC) platelet function testing currently available. Reproduced with permission from ref. 34. Fig. 1 (Springer).

Extracellular trapping by neutrophils (NETs) has been found in individuals with atherosclerosis as well as in animal models, and it has been linked to multiple potential atherosclerosis-related pathogenesis scenarios 31. Beginning with decreased activity of endothelial cells, plaque break and thrombosis due to atherosclerosis, NETs have an effect. In addition to serving as a scaffold for cells and coagulation factors, NETs also stimulate platelets, endothelial cells, and antigen-presenting cells; they also increase the expression of coagulation variables and set off the proinflammatory responses, all of which contribute to their presence in plaques and thrombi (Fig. 1IA) 32. They contribute to the aetiology of thrombosis and atherosclerotic plaques. In contrast to other cell types network topologies, NETs are almost exclusively created during the acute phase of thrombosis, when the disease is at its most dangerous. Reduced plaque stability is another consequence of NETs-induced cell death in smooth muscle. Moreover, NETs augment their role and speed up the progression of atherosclerosis by encouraging the aberrant stimulation of macrophages and upregulating the range of IL-8 and inflammasomes.

Studies have demonstrated that neutrophil infiltration is more significant in erosion-prone lesions, although macrophages are more numerous in lesions that are prone to rupture. Oxidised low-density lipoprotein (oxLDL) is a molecule utilized to induce atherosclerosis because of its propensity to concentrate in macrophages. Multiple reports demonstrate that it promotes NETs formation by neutrophils. Deleting peptidyl arginine deiminase-4 (PAD4) specifically decreases NETs production and greatly reduces macrophage-induced inflammation, and atherosclerosis. A hypercoagulable blood condition, retarded venous blood flow, and venous intima damage are the main reason for venous thrombosis, as opposed to endothelium rupture, which is the source of arterial thrombosis (Fig. 1IB) 32. Pregnancy, chronic venous blood flow, and prolonged inactivity are all associated with irregular venous blood supply and a higher risk of developing DVT. There is a high concentration of fibrin and RBCs in venous thrombosis, as well as extensive infiltration by white blood cells. There is no denying the importance of NETs extracellular traps in the study of thrombosis and haemostasis. NETs influence either arterial or venous thrombotic disorders, and they regulate thrombosis in many ways (Fig. 1II) 32. The geographical and temporal dynamics of NETs-driven thrombus development and maturation will undoubtedly be better understood owing to future research. New antithrombotic medicines that make use of this knowledge will greatly benefit. Degradation and disassembly of NETs in thrombi via pharmacological means, promoting acute thrombosis. Strategies to minimize thrombogenicity by reducing the creation of NETs may also be useful in preventing thrombosis. To completely know the potency and safety of targeting NETs in thrombosis, further preclinical and clinical research is required 33.

Thrombosis is determined by several factors in arterial blood, some of which can be reproduced *in vitro* (Fig. 1III). While thrombosis can happen in a vessel that is otherwise healthy, most local thrombus development (as opposed to embolization) takes place in arteries that have been damaged by atherosclerosis. The growth and stability of a thrombus depend on many factors, including cellular components, plasma proteases, specific elements of coagulation and fibrinolytic pathways, and blood flow parameters. The gold standard for measuring thrombosis should include as many of these characteristics as possible to replicate the situation in living organisms 34.

Stasis, endothelial damage, and hypercoagulability are the "triad" of risk factors for deep vein thrombosis (DVT) postulated by Rudolf Virchow in the nineteenth century, with platelets participation being added more recently. It has been hypothesized that adhesion of leukocytes and platelets is facilitated initially by mechanical activation of the venous endothelium, and then by inflammation-induced upregulation of surface P-selectin 35. Hypoxia increases the expression of endothelial adhesion molecules and decreases the anticoagulant effect of the endothelium surface, both of which are because of a reduction in blood flow. The coagulation cascade is initiated when activated leukocytes adhere to a surface and release tissue factor 36.

While thrombus formation is beneficial in that it assists in stopping excessive blood loss from a damaged vessel, it can also lead to dangerous clotting of the blood (thrombosis), which can give rise to disorders such as stroke, heart attack, pulmonary embolism, etc. It is generally known that platelet adhesion and aggregation are crucial in the development of a thrombus. When a blood artery is injured, endothelial cells produce von Willebrand factor (vWF) into the plasma and the subendothelial extracellular matrix 37. The vWF functions as a ligand, binding to the platelet membrane receptor GP Ib. Platelets adhere to the subendothelial matrix as a result of the formation of a GP Ib-vWF molecular connection. GP IIb/IIIa receptors can be expressed on platelet membranes, and this gap is frequently linked by a plasma protein called fibrinogen (Fg) 38. A number of the essential ligands and receptors that facilitate the adhesion and aggregation of platelets, along with major signaling pathways utilized by these receptors to monitor platelet adhesion and aggregation, are being recognized using experimental attempts to understand the molecular pathways that underlie platelet adhesion and aggregation. Platelet aggregation can be aided by the GP Ib receptor's binding to vWF, while GP IIb/IIIa receptors can aid in platelet adhesion via vWF 39. There is a growing body of evidence suggesting that shear stress caused by blood flow plays a vital role in the formation of adhesive and aggregate connections. However, when shear stress is elevated, as in microvessels or stenotic arteries, platelets adhere largely to vWF, and vWF may also influence platelet aggregation. When shear stress is moderate, as in veins and bigger arteries, platelets attach primarily to collagen and fibronectin 40.

## 3. Thrombosis in animals: an experimental model

Research into the pathogenesis of thrombosis or the effectiveness of anticoagulant medications requires the use of animal models. The following research reveals what exactly makes up thrombi in blood vessels.

### 3.1. Studies on arterial thrombosis in animals

In order to better understand cardiovascular disorders and find treatments, research into arterial thrombosis, or the development of blood clots within arteries, is essential. Our understanding of arterial thrombosis's underlying causes, risk factors, and therapeutic approaches has been greatly enhanced by research conducted in animal models 41. Mice, rats, rabbits, and other non-human primates share many anatomical, physiological, and genetic similarities with humans, making them ideal animal models for studying arterial thrombosis. Blood components, endothelial function, and platelet activity are just a few of the many elements that can be better understood with the use of these models, which provide light on the intricate processes involved in clot formation. It is common practice in research to simulate risk factors for thrombosis by manipulating blood flow patterns, creating artificial artery injury, or introducing targeted genetic mutations. Scientists are able to study the complex interaction of elements by causing controlled clot formation in these animal models. For instance, the functions of certain receptors or proteins in thrombosis have been shown by studies employing genetically engineered mice with changed clotting pathways 42. Research has revealed important molecules that play a role in the coagulation cascade, endothelial function, and platelet activation, which could be therapeutically targeted. Researchers can also evaluate the safety and effectiveness of new anticoagulant or antiplatelet treatments using animal models. Important information regarding possible adverse effects, dosage, and overall efficacy can be gleaned by inducing clot formation in these animals and then monitoring the impact of experimental medications or therapies 43. Obesity, diabetes, hypertension, and hyperlipidemia are risk factors for arterial thrombosis, and animal models are essential for researching this condition. These models shed light on the pathophysiology by showing how these factors affect the onset and course of thrombotic episodes. Animal models are helpful for study, but it's vital to remember that they aren't without their limitations 44. There are species-specific variations in physiology and treatment responses, and the results of animal studies may not always be applicable to human clinical situations. For the purpose of learning more about arterial thrombosis and creating methods to treat and prevent it in humans, these models remain crucial 45.

In a study, Steele et al, investigated the balloon angioplasty in pig model. From one hour to sixty days following angioplasty, the pathophysiological reaction to the procedure was studied in 38 normal pigs that had been heparinized. Quantification of the ^111^In-labeled platelet deposition, together with histological and electron microscopy inspection, were employed in this investigation. After one hour, the following data were observed: all arteries had complete endothelial denudation, there was a significant amount of platelets, seven out of ten pigs had mural thrombus, and nine out of eighteen arteries had a medial rip that extended into the internal elastic lamina. There was significant platelet deposition and no mural thrombus in any of the nine arteries that had tears; in contrast, all nine arteries that had no tears had severe mural thrombus. After 24 hours, it was clear that the cells lining the middle of the muscle had died. After 24 hours, platelet deposition was still considerable, but it dropped significantly to after 4 days, when some endothelium or periluminal lining cells had partially regrown. Even while the endothelial cell type of regrowth was nearly complete at 7 days, there was still no significant platelet deposition. Complete thrombotic occlusion occurred in four of the 38 pigs, with intimal proliferation of smooth muscle cells being modest and patchy at 7 days, considerably greater and more uniform at 14 days, and unaltered at 30 and 60 days following angioplasty. Histological analysis revealed that the formation of mural thrombus was the cause of a substantial stenosis that persisted 30 days following angioplasty 46.

### 3.2. Studying venous thrombosis in animals

Platelets, red blood cells, WBCs, fibrin, and neutrophils make up a day-old thrombus formed after stenosis of the rat inferior vena cava, as shown by McGuinness et al. Although monocytes clustered near the periphery of the thrombus at first, they spread outward as the thrombus matured. In a rabbit venous thrombosis model, it was found that RBC hyperaggregability brought on by pluronic F98-treated RBCs correlates with the occurrence of thrombosis 47. One common clinical issue that can lead to serious complications or even death is venous thrombosis (VT). The cornerstones of VT treatment include anticoagulation and thrombolytic treatments, that are pharmacologic or pharmacomechanical. Better VT-tailored diagnosis and treatment will be possible if the biology of thrombosis is understood. The development of new therapeutic or preventative adjuncts for VT management or prevention can be better understood with the use of in vivo models of thrombosis 48. Understanding the complex causes, risk factors, and therapeutic methods of venous thrombosis has been greatly advanced by conducting investigations in animal models. Similar to human venous thromboembolism (VTE), these models, which range in size from mice to primates and beyond, mimic the process of blood clot formation in veins by acting out scenarios including endothelial injury or stasis. Researchers learn more about how clots develop and how they dissolve when they subject veins to techniques that cause clotting, such as restriction or damage. The molecular pathways involved in venous clotting have been uncovered by these models, which have uncovered the roles of blood components, endothelial activities, and inflammatory responses 49. These models not only help us understand the basic processes, but they also act as testbeds for treatments that could be used to treat VTE. By testing the efficacy and safety of medications that target coagulation or inflammation, scientists can develop new therapies. In addition, animal models are useful for investigating the role of obesity and immobility as risk factors for VTE 50. This research provides valuable insights into the factors that raise the risk of venous clots and can help inform efforts for prevention. Because of inherent physiological differences between animals and humans, it is not always possible to extrapolate results from animal studies to human therapeutic settings. However, these models continue to be extremely helpful in guiding our knowledge and approaches to venous thrombosis management 51.

In a study, Aghourian *et al.* developed a mouse model of venous thrombosis by utilising the Vevo 770®, a micro-imaging high‐frequency ultrasound system (HFUS), for detection purposes. In order to induce thrombi in the inferior vena cava (IVC) of C57Bl/6NCr mice, researchers used two distinct thrombosis models: (i) ligation and (ii) ferric chloride (FeCl3) administration. Afterwards, HFUS was used to evaluate venous thrombosis. In both models, HFUS measures showed a positive correlation with clot pathology measurements. A thrombus forms within one hour following an IVC ligation or damage caused by FeCl_3_, and the clot's size grows for up to twenty-four hours thereafter. In particular, we show that HFUS may be utilised to track the anticoagulant dalteparin's efficacy all the way to thrombus resolution. The results demonstrate that HFUS is a non-invasive and dependable method for evaluating venous thrombosis in mice. A better knowledge of the pathophysiology of venous thromboembolism can be achieved by developing a mouse model of thrombosis employing more accurate and clinically more relevant procedures, such as ultrasonography 52.

### 3.4. Selecting a thrombosis hypothesis

The animal models for both artery and venous thrombosis provide prospects for the advancement of novel diagnostics and therapeutic techniques by revealing important details about the composition and structural characteristics of thrombi that are comparable to those of human thrombi. The timing, makeup, and structure of the thrombus that forms can be influenced by various thrombosis induction strategies, but no one model can account for all of these elements at every stage of the disease 53. Animal thrombosis models show that, in contrast to human patients, thrombus development happens most frequently in healthy arteries that have had an acute injury. It would be helpful to develop new models that more accurately depict a sick environment (such as inflammation and metabolic disorders) so that thromboembolism and thrombus shape can be better characterized 54. Because of the relative accessibility of genetic changes, small models (such as mice) have proven their worth for gaining mechanistic insights into thrombosis. However, future studies on bigger animal models that are physically more comparable to humans (especially the vasculature) may be of interest. As a result, it is crucial to choose thrombosis models and kinds of animals in accordance with the goals of the individual studies because each model has distinct advantages and disadvantages 55.

## 4. Nanoparticles as a probe for thrombus imaging and therapy

Thrombus molecular imaging first appeared in the 1970s using technetium-99m-labeled fibrinogen and iodine-131-labeled anti-fibrin antibody as gamma scintigraphy techniques 18, 19. Due to nuclear medicine's poor sensitivity and specificity, low clot blood backdrop, and unfavorable pharmacokinetics, the different nuclear medicine techniques have not been successfully used in clinical settings. However, the many targeting strategies employed by researchers in nuclear medicine are still utilized with other modalities (table 1). Molecular MRI provides the greatest potential for thrombus characterization when compared to other modalities. In contrast to ultrasonic or optical approaches, there is no ionizing radiation used in deep tissue imaging, and its spatial resolution is significantly greater (sub-millimeter) than that of nuclear imaging. A contrast agent's molecular data can also be superimposed to give context to the intrinsic anatomical picture. In addition, MRI of several image weightings can shed light on the complex plaque's composition 20-22. The disadvantage of MRI is that, as compared to nuclear methods, the sensitivity of contrast agent detection is lower. Therefore, the selection of a target while designing a molecular probe is an important aspect. Numerous NPs systems are being researched concurrently to see how they may be used in molecular imaging, with many of the applications being used to detect or treat cancer 9. For a successful delivery to the intended target, particle charge, size, shape, and hydrophilicity continue to be among the most crucial features of NPs. In-depth research has been done on polyethylene glycol (PEG) molecules as a reliable way to give hydrophilic "stealth" qualities, which frequently result in less non-specific adsorption of serum proteins *in vivo* and longer circulation durations 10. On the other hand, positively charged NPs are intended to improve endocytosis or phagocytosis for labeling cells 11. Studies are currently being conducted on a wide variety of NPs, including those made of solid lipids, micelles, liposomes, nanotubes, quantum dots, dendrimers, polymeric NPs, metallic NPs, and iodinated NPs.

### 4.1. Magnetic resonance for thrombus imaging and therapy

 A non-invasive diagnostic method called magnetic resonance imaging (MRI) can observe a few processes at the cellular or subcellular level. The magnetic resonance imaging method is based on how protons interact with one another and the molecules around a tissue of interest. Protons process or rotate at a certain frequency when brought in a strong magnetic field, and they may absorb energy from a radiofrequency pulse provided at this rotational or resonance frequency.

**Figure 2:**
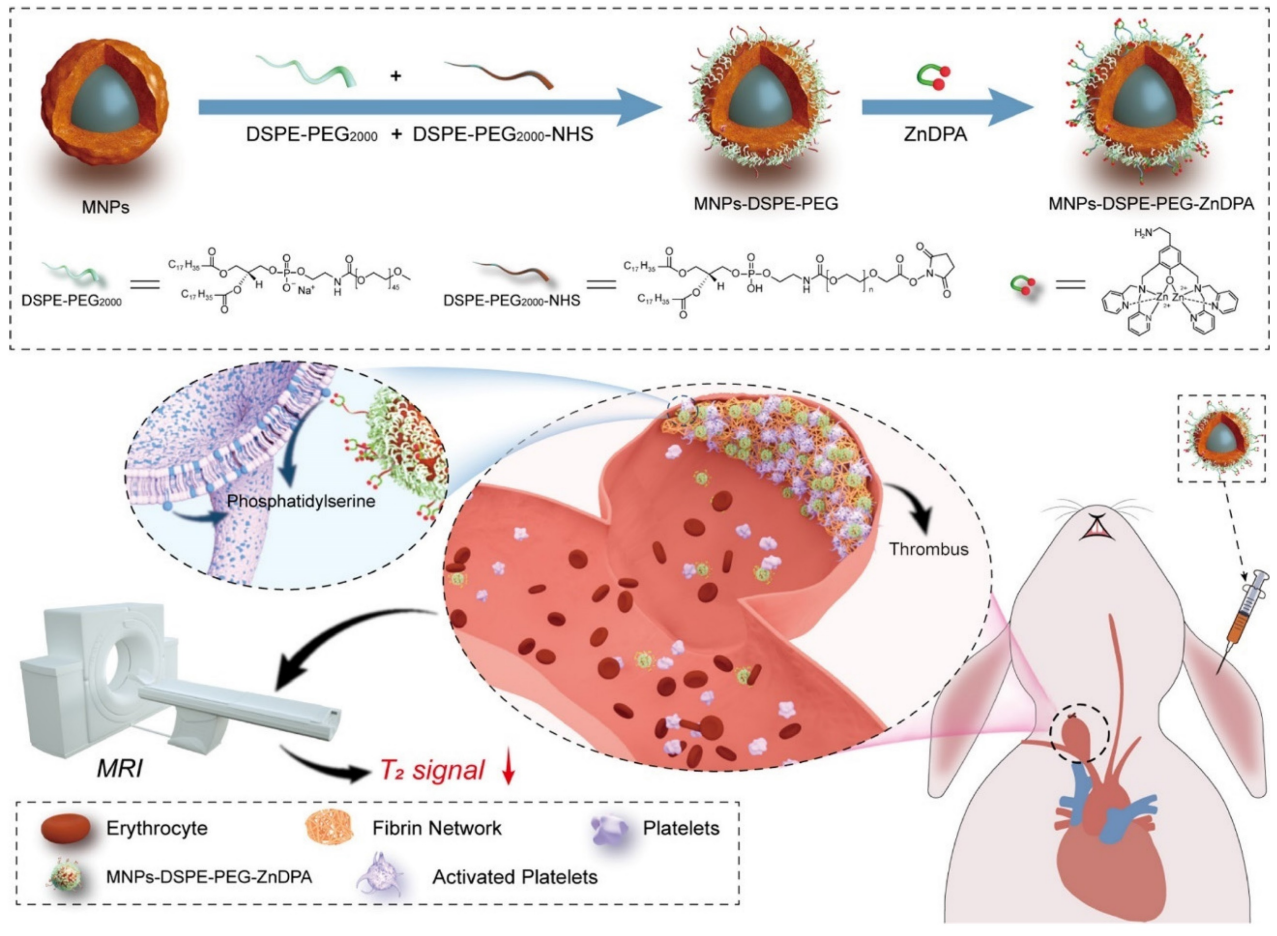
An MRI of a thrombus related to aneurysms can be improved using MFe_2_O_4_-ZnDPA NPs, as shown schematically in the fabrication process. Reproduced with permission from ref. 84. Scheme 1 (ACS Publication).

Two relaxation constants— longitudinal relaxation time (T1) and transverse relaxation time (T2)—describe the behavior of the energy injected into the system 82. Many nanoprobes have been created as contrast agents for thrombotic diagnostics owing to the quick development of nanotechnology and imaging methods. For example, Nivorozhkin *et al.* produced a trilysine-protected Gd^3+^-DTPA (gadolinium-diethylenetriamine penta-acetic acid) complex that, upon lysine cleavage by carboxy peptidase B (it is a thrombin-activatable fibrinolysis inhibitor), would have improved binding to human serum albumin (HSA). The ability to image carboxypeptidase B by MRI was made possible by the binding of the Gd^3+^-DTPA complex to HSA, which resulted in a two-fold increase in R1 relaxivity 56. In addition, Miserus *et al.* synthesized a novel bimodal α -antiplasmin-based contrast agent (CA) (Bi-α AP-CA) for the detection of early thrombus formation by using molecular MRI. This bimodal was synthesized through the coupling of Gd-DTPA and rhodamine to an α -antiplasmin-based peptide 57. In another study, Myerson *et al,* developed using a powerful thrombin inhibitor that was combined with a colloidal NPs. The therapeutic effect of this drug was both long-lasting and localised, owing to the multivalent thrombin-absorbing surface of the particle. The perfluorocarbon-core nanoparticle structures were coated with PPACK (Phe[D]‐Pro‐Arg‐Chloromethylketone) via a covalent bond. *In vitro*, thrombin activity on a chromogenic substrate was used to evaluate PPACK and PPACK nanoparticle inhibition of thrombin. Prior to acute photochemical injury of the common carotid artery, the antithrombotic activity of PPACK, heparin, non-functionalized NPs, and PPACK NPs was evaluated in living organisms by means of intravenous (i.v.) administration. The retention of perfluorocarbon particles in the carotid arteries of injured mice was evaluated using 19F MRS and 11.7 T MRI. The systemic effects of the PPACK NPs were assessed at different periods following injection using activated partial thromboplastin time (APTT) assays. An optical experiment confirmed that the anti-thrombin activity of PPACK NPs was higher than that of PPACK itself. The *in vivo* acute artery thrombosis model showed that PPACK NPs were more effective in preventing thrombosis than heparin. The precise binding of PPACK NPs to areas of acute thrombotic damage was confirmed by 19F MRS. Within 20 minutes of injecting PPACK NPs, APTT returned to normal. PPACK NPs offer a novel approach to controlling acute thrombosis locally by presenting thrombin-inhibiting surfaces at sites of newly formed thrombi. These surfaces maintain local clot inhibition even as systemic effects quickly fade 83. Further, Zhang et al, developed nanoplatform for thrombus targeting and imaging in rabbits using MRI. It consists of Zn0.4Co0.6Fe2O4@Zn0.4Mn0.6Fe2O4 NPs for imaging and Zn(II)-bis(dipicolylamine) (ZnDPA) for thrombus targeting. The biocompatibility of MFe_2_O_4_-ZnDPA NPs with a high MRI transverse relaxation time (T2) is mainly demonstrated by in vitro investigations that assess platelet safety. Using MRI and Fe quantification assays, MFe_2_O_4_-ZnDPA NPs could potentially target a thrombus by taking advantage of the unique interaction between ZnDPA and phosphatidylserine seen in active platelets within the thrombus. In addition, MRI scans taken from rabbits with common carotid artery aneurysm models reveal that MFe_2_O_4_-ZnDPA NPs might build up in the aneurysm-related thrombus within the first fifteen minutes of injection and then diminish within the subsequent forty-five minutes. However, MFe_2_O_4_-ZnDPA NPs have the potential to improve the aneurysm's outline by reducing the MRI T2 signal of the thrombus associated to the aneurysm. The results of this study show that nanoplatforms can improve the identification of intracranial aneurysms and thrombus associated with them, which can aid in the treatment of these conditions (Fig. 2) 84. In another study, Ta *et al.* synthesized a dual contrast iron oxide nanoparticle (DCIONs) for magnetic resonance imaging using co-precipitation at high temperature. To facilitate targeting, single-chain antibodies (scFv) directed against active platelets were added to DCIONs 59. To develop novel fibrinolytic substances that target thrombus using NPs' multifunctional theranostic properties; this could lead to the creation of effective thrombolytics with minimal harmful side effects. Statistical study revealed that the targeted thrombolytic nanoagent bound to fresh-frozen plasma clots more effectively than the control nanoagents (p < 0.05). In vitro fibrinolytic activity against human plasma clots was found to be similar for targeted, control, and free tPA samples when normalised by S2288-based amidolytic activity, as confirmed by ELISA D-dimer tests. As shown by intravital fluorescence microscopy, the fibrinolytic nanoagent that was targeted to FXIIIa effectively bound the edge of intravascular thrombi. Studies on fibrinolysis in living organisms found that the FXIIIa-targeted drug was just as effective as free tPA in lysing pulmonary emboli (p > 0.05). Thrombolytic nanoagents that target FXIIIa have shown promise in both laboratory and animal studies for the treatment of thromboembolism. They intend to conduct more research into this class of nanoagents to learn more about their safety and general effectiveness 85. Also, Ta and colleagues developed an entirely novel nanosensor for thrombus detection and aging. On a thrombin cleavable peptide, iron oxide nanoclusters (IONCs) were functionalized with Gd-DTPA (diethylenetriamine penta-acetic acid gadolinium (III) dihydrogensalthydrate). Further labeling of Gd-DTPA with a dye containing a fibrin-binding peptide was done in order to target both new and old thrombi. Gd-DTPA enhances the T1 (spin-lattice relaxation) contrast in MRI, but IONCs have an enhanced T2 (spin-spin relaxation) impact 61.

Moreover, Wang *et al.* outline a novel approach to imaging active platelets utilising state-of-the-art fluorine-19 (19F) MRI. The active conformation of integrin glycoprotein IIb/IIIa (GPIIb/IIIa; αIIbβ3, CD41/CD61) was selected as the target for selective targeting of activated platelets. The outcome of the study show that TargPFCs produce the best contrast results in both mice and humans when using background-free molecular 19F MRI to examine active platelets. In both laboratory and living organism studies, TargPFCs only collect at locations where platelets are activated because they bind only to activated GPIIb/IIIa receptors. In order to enhance patient outcomes, it is crucial to diagnose activated platelet-involving disorders (thrombotic, atherosclerotic, inflammatory, and malignant) as soon as possible. This allows for early treatment intervention. Although this is only a proof-of-concept study, the utilisation of human scFvs with low antigenicity and well-tolerated PFCs, in conjunction with background-free 19F MRI, provides significant evidence for the potential of early identification of many disorders that could have far-reaching health consequences 62.

### 4.2. Ultrasound for thrombus imaging and therapy

**Figure 3:**
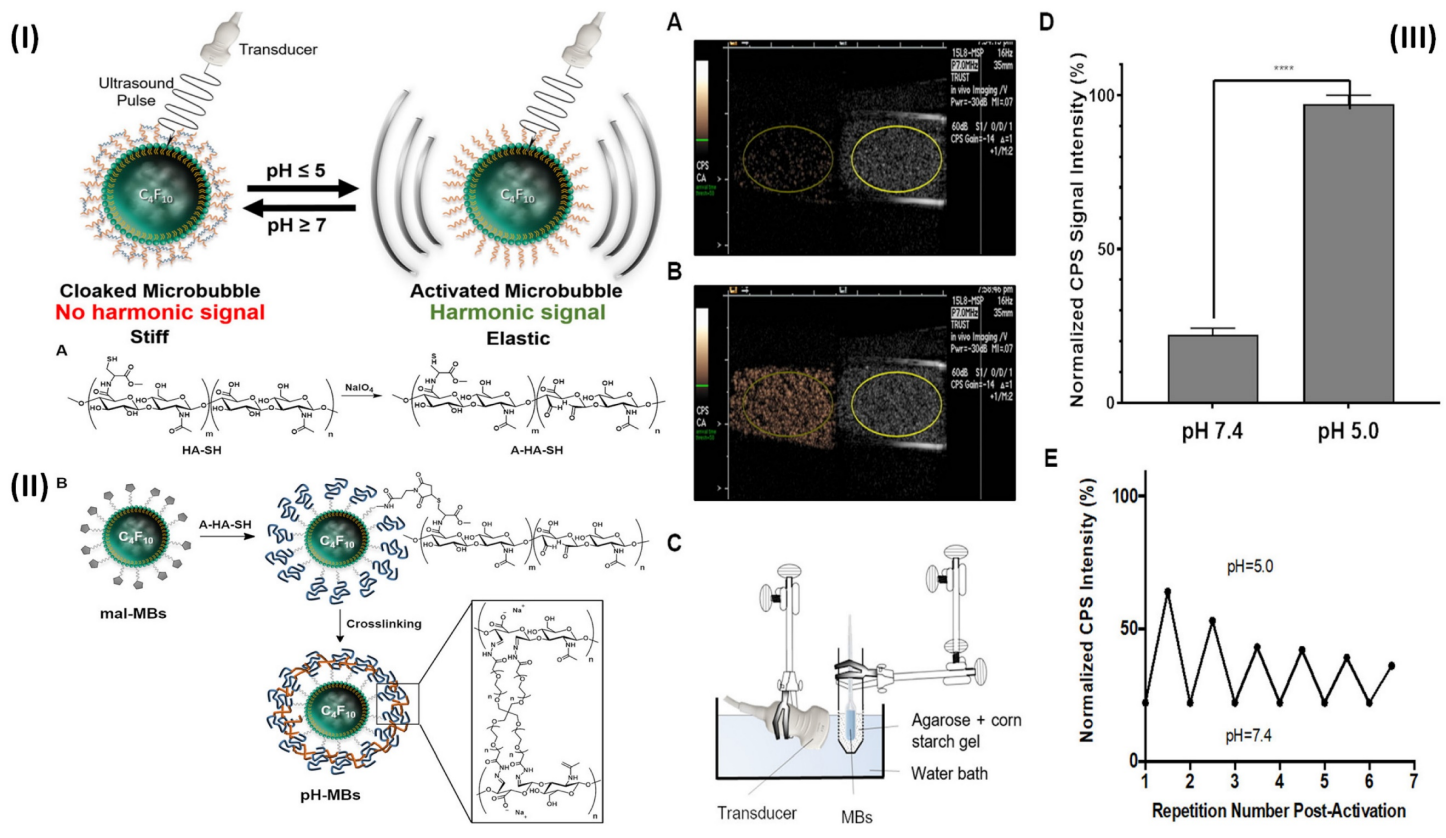
(I) Schematic representation of the production of pH-MBs shows the reversible activation of pH-sensitive microbubbles. (II) The diagrammatic representation of the conjugation of A-HA-SH to mal-MBs and subsequent cross-linking with a pH-sensitive cross-linker to create pH-MBs; (III) pH-MBs imaged with CPS (left) and B-Mode (right) (A) at pH 7.4 and (B) at pH 5 using the *in vitro* setup shown in (C). (D) Peak CPS signal SD of pH-MBs in neutral (pH 7.4) and acidified (pH 5) conditions (n = 3). The standard deviation is shown by error bars. (E) After initial activation, the activation reversibility of pH-MBs was studied when cycling between pH 5.0 and 7.4. Reproduced with permission from ref. 64. Fig. 3, and Fig. 5 (ACS Publication).

 A transducer in ultrasonography (US) transmits high-frequency sound waves through the body. Some of the energy of a transmitted ultrasonic pulse is reflected to the transducer when it strikes a tissue target. The depth of the target in the transducer beam is determined using the time of flight of this ultrasonic echo. To create images, pulse-echo properties such as echo amplitude, target spatial position, and frequency shift between the delivered pulse and the received echo are all used 86, 87. In a study, Li *et al.* prepared fucoidan-MBs targeted ultrasonic contrast material that is made of biodegradable poly (isobutyl cyanoacrylate) and functionalized with fucoidan to scan venous thrombus. The PIBCA (polysaccharide-coated poly (isobutyl cyanoacrylate) microbubble was created using the polymerization process. One of the polysaccharides, fucoidan, was thought to functionalize the MBs in particular because of its strong affinity for P-selectin. Fucoidan-MBs specifically bind to P-selectin produced by human-activated platelets during venous shear stress, in contrast to anionic carboxymethylated dextran MBs. Using the ferric chloride (FeCl_3_) produced inferior vena cava (IVC) non-occlusive thrombus rat model, the *in vivo* targeting of Fucoidan-MBs to P-selectin overexpressed on thrombus was assessed. Colour Doppler Flow Imaging verified the existence of venous blood flow around thrombi, while B-mode examined for non-occlusive thrombi. Verification of bound Fucoidan-MBs in the thrombus region was done accurately ten minutes after injection. After MBs burst by destructive pulse, the signal intensity of the binding area in the thrombus was noticeably reduced. There was a considerably larger accumulation of Fucoidan-MBs compared to CM-Dextran-MBs, as measured by subsequent quantifications of the decreased signal intensity in the thrombus area before and after destructive pulse. In addition, the inferior vein walls of healthy rats did not show any Fucoidan-MBs. Furthermore, no adverse effects were observed in the healthy rats who were injected with MBs; furthermore, they were found to be living several months later 63. In another study, Wang *et al,* developed theranostic NPs (CyBA/PFM-NPs), that enhance photoacoustic contrast when activated by hydrogen peroxide (H_2_O_2_) and have antithrombotic properties. The fucoidan section within the carrier of CyBA/PFM NPs was engineered to target platelet-rich clots. When activated by H_2_O_2_, it produces fluorescent "CyOH" molecules, which activate the photoacoustic signal. The fluorescence of CyBA/PFM NPs was significantly amplified after incubation with new clots, indicating that they effectively scavenged intracellular ROS. Using a mouse model of carotid thrombosis caused by FeCl3, the researchers found that CyBA/PFM NPs greatly enhanced the photoacoustic contrast in thrombogenic tissues, efficiently neutralised ROS at the occlusion site, reduced thrombus formation, and normalised the soluble CD40L level while doing so. The promising imaging capabilities, robust antithrombotic effects, and tolerable biosafety of CyBA/PFM NPs give them great promise as nanoscale theranostics for cardiovascular disorders linked to H_2_O_2_ 88. Further Burns *et al,* developed a targeted and activatable MB platform, a brand-new pH-sensitive ultrasonic contrast agent that was tested *in vitro* to see how it would respond to acidic conditions. This activatable MB platform, which is adaptable and can be modified to incorporate a variety of biological markers, like enzymes, reactive species of oxygen, and redox conditions as biological markers of choice, serves to illustrate the efficacy of this strategy. Altering the crosslinker to something like an enzymatically degradable peptide can immediately alter the MB platform's functionality. Hyaluronic acid (HA) polymer was attached to the phospholipid membrane by site-specific and bio-responsive MBs. Cross-linking HA makes the shell more rigid, transforms it into a hydrogel, and dampens the harmonic signal. Dampening MB oscillations to silence their signal and preserve it when they encounter the preferred indicator can improve the detection and specificity of illnesses like deep vein thrombosis (DVT) 89. By fusing the biocompatible hyaluronic acid (HA) polymer with the phospholipid shell, they created MBs that are both targeted and bio-sensitive. HA hardens the shell and muffles the harmonic signal when it is cross-linked, giving it the characteristics of a hydrogel. Employing a reversible pH-sensitive cross-linker, they verified and validated their platform in this experiment. (Fig. 3I). Although HA was chemically changed, the pH-MBs still targeted HeLa cells (Fig. 3II). This finding reveals that oxidation and crosslinking do not diminish HA's capacity to target CD44. This was to be predicted because CD44 has a small binding site on HA. This study used a diagnostic ultrasound scanner with CPS nonlinear harmonic imaging to take pictures of 3 x 10^4^ pH-MBs in a portable pipette suspended in 3 mL phosphate buffer solution 1X at pH 7.4 (Fig. 3IIIA). The B-mode picture was displayed alongside the MB-specific CPS image. Scans were performed in real-time using ultrasonography as the material was acidified to a pH of 5 (Fig. 3IIIB). They measured the CPS signal strength in neutral and acidic pH-MBs by drawing region of interest (ROIs). The CPS signal was found to rise by a factor of five when acid was introduced (Fig. 3IIID). The harmonic signal was not altered by the acidification of non-cross-linked HA-MBs. The shift in harmonic signal after acidification is mainly related to higher flexibility because there was no noticeable change in pH-MB size distribution between before and after acidification. They used NaOH to neutralize the pH to establish that this platform is reversible since the hydrazone linkages are reversibly broken by acid. After numerous cycles of acidification and neutralization, they saw that the CPS signal strength reverted to its pre-acidification level, and reacidification restored the reduction in CPS signal. Peak CPS intensity was measured before and after each cycle, and it was found that there was a slight decrease in peak intensity after each cycle (Fig. 3IIIE). This was probably caused by MB instability by NaOH, progressive MB dilution, and most importantly MB degradation by ultrasound over more than 30 minutes of continuous instantaneous imaging 64.

Recently, Vishnu et al. developed NK-loaded (NK-LS) non-targeted and targeted (RGD-NK-LS, AM-NK-LS) liposomes via reverse phase evaporation. Physiochemical characterization confirmed successful synthesis and surface conjugation. Targeted liposomes exhibited enhanced platelet affinity, demonstrated potent antithrombotic efficacy *in vitro*, and showed increased thrombolysis *in vivo* without bleeding complications. The effectiveness of the liposomes was evaluated using real-time Doppler imaging on the carotid artery. Rats received various treatments (saline, NK, NK-LS, RGD-NK-LS, AM-NK-LS) when blood flow decreased due to thrombus formation. After 30 minutes, ultrasound imaging showed recovery in blood flow. Percentage thrombolysis, indicating blood flow restoration, was: saline 0.89%, NK 19.81%, NK-LS 24.49%, RGD-NK-LS 75.5%, and AM-NK-LS 72.62%. This demonstrates the significant efficacy of RGD-NK-LS and AM-NK-LS in restoring blood flow post-thrombus formation 90.

### 4.3. Computed tomography for thrombosus imaging and therapy

 The method of creating cross-sectional pictures using X-rys and computers is called computed tomography (CT). The ROIs is traversed by many slices or photographs, most of which are continuous. Then, each image is examined separately to check for anomalies. It's mandatory to keep in mind that anatomical structures in a CT picture are not immediately shown, as in regular X-ray images 91. The colors black, white, and various degrees of gray (referred to as the gray scale) are applied to small squares (also known as picture components or pixels) that are organized in columns and rows to create the CT image (a matrix). A chunk of tissue is represented by each pixel (volume element or voxel). Each pixel's color in the grey scale is determined by the type of tissue that the X-ray beam penetrated in that slice 92.

In a study, Cai* et.al.,* prepared gold nanocrystals to form colloidal AuNPs. Due to their composition and relatively larger size, NPs demonstrated a longer retention duration in the blood arteries. Due to its significant potential for application as a blood-pool contrast agent, this AuNPs-PEG also implies possible implications for CT perfusion imaging 65. In another research work Kim *et.al.,* developed a nanoprobe for CT-based direct cerebral thrombus imaging. The fib-GC-AuNP, glycol-chitosan-coated gold NPs (GC-AuNPs) were created and coupled to fibrin-targeting peptides. Fib-GC-AuNPs had a greater ability to bind fibrin than GC-AuNPs, and on microCT (mCT), thrombi were visible as having a high density. The rapid identification and assessment of cerebral thrombi as well as the tracking of the tPA-mediated thrombolytic impact, which mirrored the histopathological outcome of the stroke, were made possible by the use of fib-GC-AuNP in mCT imaging 66. Recently Koudrina et al, formulated a method for aptamer functionalized core-shell NPs (CSN), or superparamagnetic gold-coated iron-oxide NPs, to target the molecular components of thrombi (Fe_3_O_4_-AuNPs). First, a phantom CT scan was used to evaluate FA-Fe_3_O_4_-AuNPs' capacity to create noticeable contrast enhancement at low nanomolar concentrations. It should be noted that at millimolar doses, the observed contrast increased at a faster rate for FA-Fe3O4-AuNPs (1697 HU mM1) than for Isovue (iopamidol) (11.3 HU mM1). This allowed for the *in vitro* testing of FA-Fe3O4-AuNPs. Treatments with FA-Fe3O4-AuNPs were compared to those with FB139-Fe3O4-AuNPs, Isovue, and PBS, but no noticeable or statistically significant difference was seen. It follows that the CT contrast was not produced by the gold mass ratio, and that future studies will most likely require a far thicker covering 67*.*

### 4.4. Near-infrared fluorescence for thrombosis imaging

Near-infrared (NIR) fluorescence is a type of optical imaging that utilizes light in the NIR ranges of the electromagnetic spectrum (usually between 700 nm and 900 nm) to visualize biological tissues and molecules. The advantage of NIR fluorescence is that it can penetrate deep into biological tissues and is minimally absorbed by water and hemoglobin, which can interfere with other imaging modalities. NIR fluorescence imaging works by using a fluorescent dye or probe that emits light in the near-infrared range when excited by a light source 93. The fluorescent dye or probe is typically conjugated to a targeting molecule such as an antibody, peptide, or small molecule that binds to a specific biomolecule of interest. When the targeting molecule binds to its target, the fluorescent dye or probe emits light, which can be detected by a specialized camera or imaging system.

**Figure 4:**
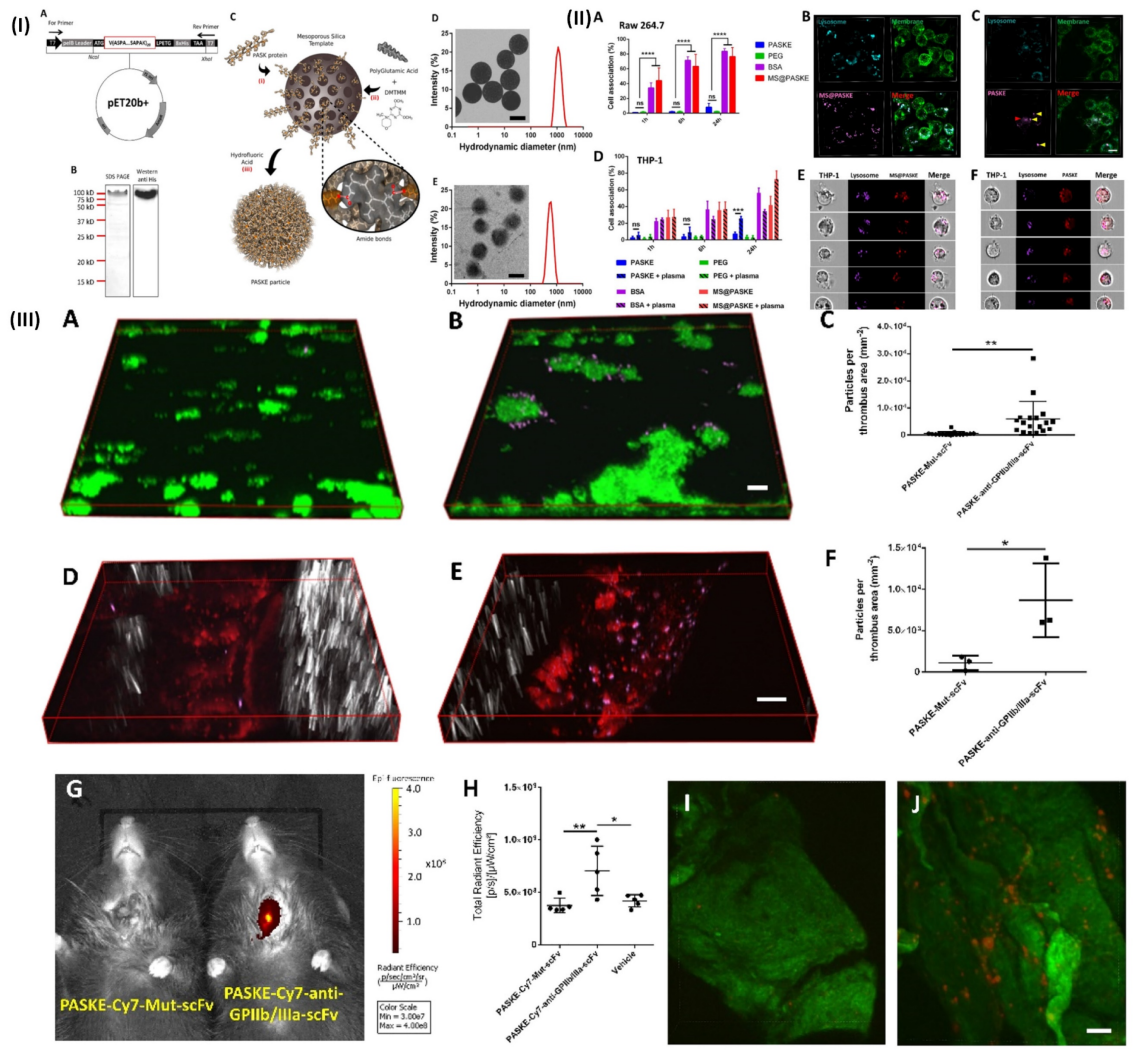
(I) (A) Vector map of the PASK protein optimized for bacterial expression 20-LPETGGLE-His8). (B) 12% SDS-PAGE and Western blot analysis using a horseradish peroxidase coupled to the anti-6XHis-tag antibody of the purified PASK protein. (C) Scheme of PASKE particle assembly. Transmission electronic microscopy (TEM) images and dynamic light scattering (DLS) for size distributions of MS@PASKE (D) and PASKE (E); (II) Cell interaction of PASKE, PEG, BSA, and MS@PASKE with human THP-1 and murine macrophages (RAsW 264.7); (III) functionalization of PASKE particles with the anti-GPIIb/IIIa scFv to impart a specific affinity to active platelets. Reproduced with permission from ref. 68. Fig. 1, Fig. 2, and Fig. 4 (ACS Publication).

NIR fluorescence imaging has several applications in biomedical research and clinical medicine, including tumor imaging, and lymph node mapping of various imaging. One of the key advantages of NIR fluorescence imaging is that it can be used to visualize biological processes in real time, providing valuable insights into disease mechanisms and treatment responses 94. For example, Bonnard *et al.* prepared a molecular imaging probe using mesoporous silica as a template, they developed a recombinant protein along with enhanced hydrophilic character owing to the proline, alanine, and serine (PAS) amino acid repeat. After that, the protein was cross-linked into particles using polyglutamic acid (E) and lysine (K). These particles show excellent results as a non-invasive tool for molecular imaging in a mouse model of carotid artery thrombosis when decorated with an anti-glycoprotein IIb/IIIa single-chain antibody that targets functional platelets and is tagged by NIRF molecules. In a 37.8 kDa protein building unit which was later processed and purified by incorporating a polyhistidine tag to the C-terminu PAS were erratically repeated. In Fig. 4IA. Through the use of the technique of western-blot anti-his-HRP and sodium dodecyl sulfate (SDS) gel electrophoresis, E. coli produced the PASK protein which was separated, purified and identified to be around a 100 kDa protein (Fig. 4IB). The large difference in estimated size is the main reason for decreased SDS binding to the remarkably hydrophilic PASK protein, which is in line with previous findings of PASylated proteins and illustrates the reason they move slower in electrophoresis. To minimize changes in nanoparticle quality between batches, they employed only SDS gel electrophoresis-verified, highly pure PASK protein building blocks. Particle formation was either inconsistent or impossible when working with less-than-pure starting materials. The separated PASK building blocks were put together into particles using the MS template method that was previously described (Fig. 4IC). To produce well-dispersed PASKE particles with a 730 nm diameter, the 1200 nm MS template was dissolved in buffered hydrofluoric acid (Fig. 4ID and E). The low accumulation of unwanted material on solid surfaces property of the PASKE particles was assessed *in vitro* using phagocytosis studies on murine macrophages (RAW 264.7) and human monocytic cells (THP-1) (Fig. 4II). Bovine serum albumin (BSA)-based particles and MS@PASKE particles were examined to evaluate the significance of the PASK protein in comparison to an extensively researched model protein. The PEG particles are seldom ever taken up by the RAW cells, whereas the silica particles are readily taken up by them (Fig. 4IIA). In comparison to PEG particles, PASKE particles display a marginally higher association signal after 24 hours. Confocal imaging demonstrates that certain PASKE particles that exhibit interaction with cells after 24 hours have not all been phagocytosed but instead are still bound to the cellular envelope (Fig. 4IIC, yellow arrows). The obtained diffused fluorescent signal (red arrow) indicates that the inhaled ones are being degraded in the lysosomes. In their experiment, human plasma was added to cell media to investigate the consequences of protein corona development, with the individual's monocytic cell line THP-1 serving as the target cells. (Fig. 4IID). PEG particles continued to associate poorly even after plasma was added, but PASKE particles displayed delayed association behavior, with the difference between the two being negligible after 1 h of incubation, but increasing dramatically after 24 h. The flow cytometry imagining was done by incubating the THP-1 cell in the human plasma for 1day, this shows that, in contrast to the MS@PASKE particles, which did not degrade over time, the phagocytosed PASKE particles were particularly confined in the lysosomes and demonstrated signs of breakdown, as demonstrated through the diffused fluorescence signal seen (Fig. 4IIE and 4IIF). To examine thromboembolic platelet activity, they developed a molecular imaging tool and explored the imaging capabilities of the PASKE particle platform. To do this, they first attached the BCN-modified anti-GPIIb/IIIa-scFv to the surface of the PASKE-N3 by copper-free click chemistry. The PASKE-anti-GPIIb/IIIa-scFv's affinity for thrombosis was examined using microfluidic channels in mice models of mesenteric artery thrombosis (Fig. 4IIID, E, and F) as well as human blood microthrombi (Fig. 4IIIA, B, and C). Compared to PASKE NPs decorated by the non-binding control scFv, Mut-scFv-functionalized PASKE particles performed more effectively in targeting the microthrombi in both experiments. The margination effect that these particles undergo lends assistance to effective targeting. Larger particles are, nevertheless, more vulnerable to shear detachment pressures. The 200 nm particles showed better binding than 12 nm particles in a flow chamber setup that mimics targeting of the vascular wall. An *in vivo* imaging system (IVIS) was used to assess PASKE-anti-GPIIb/IIIa-scFv that had been labeled with cyanine 7 NHS ester for its ability to diagnose carotid thrombosis in mice. The PASKE-anti-GPIIb/IIIa-scFv-Cy7 therapy significantly increased the carotid thrombosis signal uptake in contrast to the nontargeted (PASKE-Mut-scFv-Cy7) and vehicle control subjects (Fig. 4IIIG, H). Through the use of a near-infrared (NIR) confocal microscopy system, they were able to visually confirm that the site-specific PASKE particles had accumulated at the thrombotic carotid artery (Fig. 4IIII, J) 68. In another study, Wu *et al.* developed TTQ-PEG-c(RGD) as a newly organic NIR-II nanoprobe with great stability, minimal cytotoxicity, high targeting ability, and strong NIR-II performance. NIR-II fluorescence imaging with the electron acceptor 4,9-bis(5-bromothiophen-2-yl)-6,7-bis(4-(hexyloxy)phenyl)-[1,2,5]thiadiazolo[3,4-g] quinoxaline (TTQ) was achieved by utilizing a two-arm multifunctional telechelic glycopolymer. The probe targets active platelet GPIIb/IIIa during the early stages of thrombus formation, allowing it to successfully differentiate between an early thrombus and an older thrombus *in vivo*. It is effective against thrombosis in both animal models and lab dishes 69. In addition, Ha *et al.* developed and reported a terminal amine polyethylene glycol (PEG)- tirofiban analogues (SPS) TIRO-CyAl5.5 are attached to NIR fluorophore-labeled PEG6. This novel GPIIb/IIIa receptor inhibitor-based NIR fluorescent molecular imaging agent may bind to and detect platelets both *in vitro* using human platelets and *in vivo* using a mouse model of venous thrombosis. The probe's high TBR ratio and short blood half-life make it useful in many applications where active platelets are a sign of illness. They measured the size of the venous thrombi after injection of the agents for both the TIRO-CyAl5.5 and PBS groups to evaluate their potential therapeutic effect on thrombus formation. This is important to note because diagnostic imaging agents should not cause thrombi. TIRO-CyAl5.5 was administered while imaging at a dosage of 10 nmol/kg. Based on the results, it can be concluded that TIRO-CyAl5.5 did not have a significant anti-thrombotic effect *in vivo*, as there were no significant changes in thrombus length or area between the groups. The co-localization of the fluorescent probe to platelets was further confirmed by immunofluorescent labelling of the thrombi. On the other hand, neither the control group nor the group given free dye (CyAl5.5) had a significant accumulation of intra-thrombus, even after administering excessive tirofiban to induce blockage of GPIIb/IIIa. The imaging agent can still bind to other sites on the platelet, as shown in these blocking studies. However, because tirofiban is specific to GPIIb/IIIa, the elimination of intrathrombus accumulation suggests that TIRO-CyAl5.5 primarily binds through GPIIb/IIIa-mediated mechanisms 70. Further Chen *et al.* developed a phthalocyanine-based clot homing probe and its application to *in vivo* 3D imaging of two distinct mice thromboembolic models. The NIRF probe successfully binds to blood clots and provides information on their size as well as their position for this study's analysis. Following treatment with recombinant tissue-type plasminogen activator (rtPA), the probe may be used to objectively observe and monitor *in vivo* thrombolysis. Therefore, actual time localization, measurement, and record-keeping of embolisation and thrombolysis are provided by this NIRF probe. To track the development of the thrombolytic treatment, it can also be added to thrombolytic agents 71.

### 4.5. Photoacoustic for thrombus imaging and therapy

**Figure 5:**
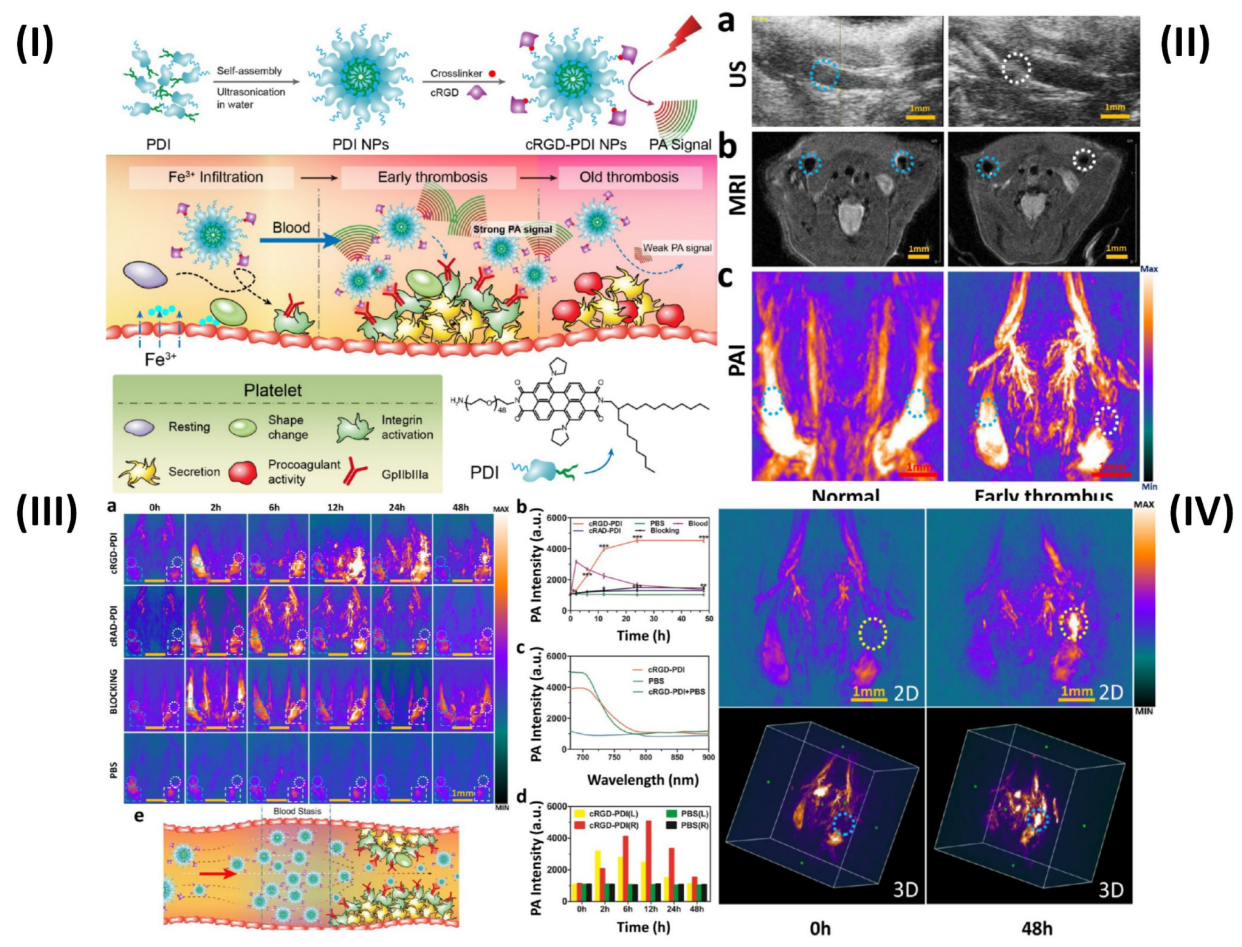
(I) Schematic illustration of the preparation of cRGD-PDI NPs and process for reducing early thrombus explicitly through PAI; (II) US, MRI, and PAI showing *in vivo* identification of early thrombus; (III) *In vivo* PA detection of early thrombus; (IV) Early thrombus PAI (area encircled by the yellow and blue dotted lines in the 2D image and 3D image, respectively) 0 hours and 48 hours following the injection of cRGD-PDI NPs. Reproduced with permission from ref. 73. Fig. 1, Fig. 5, Fig. 6 and Fig. 7 (ACS Publications).

 The photoacoustic imaging (PAI) effect, which refers to the creation of acoustic waves by the absorption of electromagnetic (EM) radiation, such as optical or radio-frequency energy, is the basic foundation for PAI. Megahertz ultrasonic waves, also known as photoacoustic or thermoacoustic signals, are often generated in biological tissues using nonionizing waves, such as brief laser or rf pulses. The goal of PAI is to combine high contrast from light, or rf, absorption with ultrasonic resolution. To light the biological sample in PAI, a nanosecond pulsed laser (pulse duration 10 ns) is frequently employed. Temperature rises as a result of the molecules' conversion of optical energy into heat 95. In a study, Ha *et al.* synthesized gold nanorods (GNRs) and used PAI technique to show the simultaneous tracking and time course analysis of adhesion molecules, ICAM-1, and E-selectin which are biomarkers of endothelial inflammation. Leukocyte adhesion molecules are linked to the inflammatory process and are crucial for the formation of thrombi 72. However, Cui *et al.* used amphiphilic PDI (perylene-3,4,9,10-tetracarboxylic diimide) and cRGD (Arg-Gly-Asp) peptide to prepare organic semiconducting NPs. The prepared cRGD-PDI NPs were capable of differentiating between early and old thrombus (Fig. 5I). Ultrasound (US) revealed a wall-adherent thrombus that appeared as a fuzzy outgrowth on the wall (in the marked white region) in the lumen that had an early thrombus compared to the typical jugular venous lumen; the early thrombus appeared white-gray in this case, while luminal blood appeared black or dark grey. The intrinsic weak contrast of the thrombus makes it challenging to distinguish it from surrounding tissues using ultrasonic technology. Since MRI offers complete anatomical reference data and greater spatial resolution than the US, the jugular vein is visible on T2-weighted MRI in Fig. 5IIb. However, similar to the earlier case, the MRI did not reveal any definitive evidence of thrombus formation in the affected blood artery. The reason for this is that MRI may not pick up a small, non-occlusive thrombus since it has such a negligible effect on blood flow. Because hemoglobin in blood absorbs NIR light at a far higher rate than that of surrounding tissues, PAI can offer high-resolution, high-contrast images of blood vessels. Even the smallest vessels' shape and location are discernible by PAI in Fig. 5IIc. The aberrant vessel with thrombus may be seen in the white circular area and can be distinguished from the regular vessel in the blue-ringed area by the lack of PA signal. The decreased PA signal of blood shows the presence of a thrombus in this area due to ischemia (a lack of hemoglobin). PAI offers promising results for thrombus identification since its ability to observe thrombus growth in comparison to MRI and US is evident. Due to the PAI's signal-off effect on the thrombus, it is more important than ever to highlight the thrombus with a PA contrast material to understand everything there is to know about the thrombus. The organic semiconducting NPs have a great affinity for the GPIIb/IIIa receptor on active platelets, a high photoacoustic intensity, and outstanding photostability and biocompatibility. Next, cRGD-PDI NPs were used to perform *in vivo* PAI of an early thrombosis state. There were three sets of mice, each containing one wall-adherent thrombus. In one group, 300 µL of 3.33 mg/mL concentrated cRGDPDI NPs were injected into the tail vein, while in the other two groups, 300 µL of cRAD-PDI NPs and PBS were administered. Fig. 5IIIa displays the PAI data across all experimental groups and times. Before the injection of NPs, all three PAIs showed a distinct signal-off impact in the thrombus areas (shown by the white-outlined area) and normal artery PA signals (shown by the blue-circled area). A portion of the thrombus began to form in the white circle zone with an amplified PA signal two hours following the injection of cRGD-PDI NPs. Fig. 5IIIa, b shows the PA signal 6 hours after NP injection, clearly amplified over the thrombus region, and 4.3-fold higher than before NP treatment. After 48 hours, the cRGD-PDI NP-induced increase in thrombus PA signals was more pronounced. Fig. 5IIIc displays an *in vivo* PA signal in the thrombus location, which peaked at 700 nm 12 hours post NP administration. The position of the PA peak for cRGD-PDI NPs after deposition in the thrombus is consistent with the typical PA signal for cRGD-PDI NPs in a solution with water but markedly dissimilar to that for cRGD-PDI NPs administered with PBS. The ability of the cRGD-PDI NPs to attenuate thrombus intensity *in vivo* demonstrates their propensity for binding to the thrombus. Mice injected with cRAD-PDI NPs and PBS, on the other hand, did not show an increase in PA signal in the thrombotic region. When comparing the cRGD-PDI NP group to the cRAD-PDI NP control group 24 hours after injection, PA strength in the thrombus site rose by a factor of 3.5 in the cRGD-PDI NP group (Fig. 5IIIb). This demonstrated the efficacy of cRGD-PDI aimed at pre-thrombus, possibly because of its high affinity to GPIIb/IIIa on activated platelets involved in pre-thrombus formation. This was shown with a GPIIb/IIIa blocking experiment *in vivo*. The GPIIb/IIIa-blocking medication eptifibatide and the cRGD-PDI NPs were administered into a tail vein an hour apart. At each time point, it was discovered that the thrombus signal region had vanished entirely. According to additional research, the normal vasculature PAIs were brightest 2 hours post PDI NP administration in both the cRGD-PDI NP and cRAD-PDI NP groups (Fig. 5IIIa, dotted blue line in square), but gradually dimmed thereafter. Fig. 5IIIa, depicted by the white line in the square, shows that the blood signal strength on the thrombus site peaked at 12 hours in both groups, much later than in the control artery (Fig. 5IIId). The temporary interruption in the flow of blood where the thrombus impeded the blood flow allows for improved deposition of cRGD-PDI NPs in the adjacent blood region, explaining the prolonged retention duration observed (Fig. 5IIIe) 73.

### 4.6. Multimodal technique for thrombus imaging and therapy

The most often utilized imaging modalities in clinics include ultrasound, positron emission tomography (PET), single photon emission computed tomography, magnetic resonance imaging (MRI), computed tomography (CT), and optical imaging. It is challenging to get precise and trustworthy information at the illness site since each imaging modality has fundamental limits, such as insufficient sensitivity or spatial resolution, in addition to its special benefits 96, 97. The specificity, resolution, and quantitative capabilities of imaging modalities differ. The main problem with a single imaging modality is the inability to guarantee the consistency of the diagnosis, which is essential in deciding the course of therapy. Multimodal theranostics can help to tackle this issue because each imaging modality has distinct advantages of its own. Multimodal imaging provides a variety of procedures with complementary capabilities to get around a modality's inherent limits 98. In a study, Uppal *et al.* generated a discrete bimodal theranostics probe with a fibrin target. At the C- and N-termini of a fibrin-specific peptide, two different imaging reporters were attached. Optical imaging, MRI, and PET imaging tests demonstrated the ability of these probes to detect fibrin over a broad range of probe concentrations. A fluorophore (OI) at one terminal and a chelator for Gd (MRI) or ^64^Cu (PET) at the other terminus might be included using a combined solid/solution-phase synthetic method 74. In another study, Ciesienski *et al.* designed a peptide probe for imaging fibrin with three novel peptide-chelate conjugates. Three fibrin-specific peptides were chelated with ^64^CuCl_2_ and conjugated as monoamides of 1,4,7,10-tetraazacyclododecane-1,4,7,10-tetraacetic acid (DOTA) at the C- and N-termini. After derivatization with 64Cu-DOTA, the fibrin affinity of all three probes was preserved. In a rat model utilizing hybrid MR-PET imaging, FBP1 and FBP2 demonstrated accurate identification of the arterial thrombus and imaging effectiveness 75. Although, Song *et al,* created a nano-agent by combining a targeting ingredient, CREKA (cysteine-arginine-glutamicacid-lysine-alanine), a peptide with a specific affinity for fibrin, with a superparamagnetic iron-oxide nanoparticle (SPION). For sensitive multimodal molecular imaging of microthrombus, it is important to utilise a high-avidity, microthrombus-specific agent with a significant paramagnetic impact and near infrared fluorescence. The targeted molecule in this investigation was the clot-binding peptide CREKA, the NIR labelling agent IR783 was used for optical imaging, and SPION was used as an imaging agent for MRI 76.

**Figure 6:**
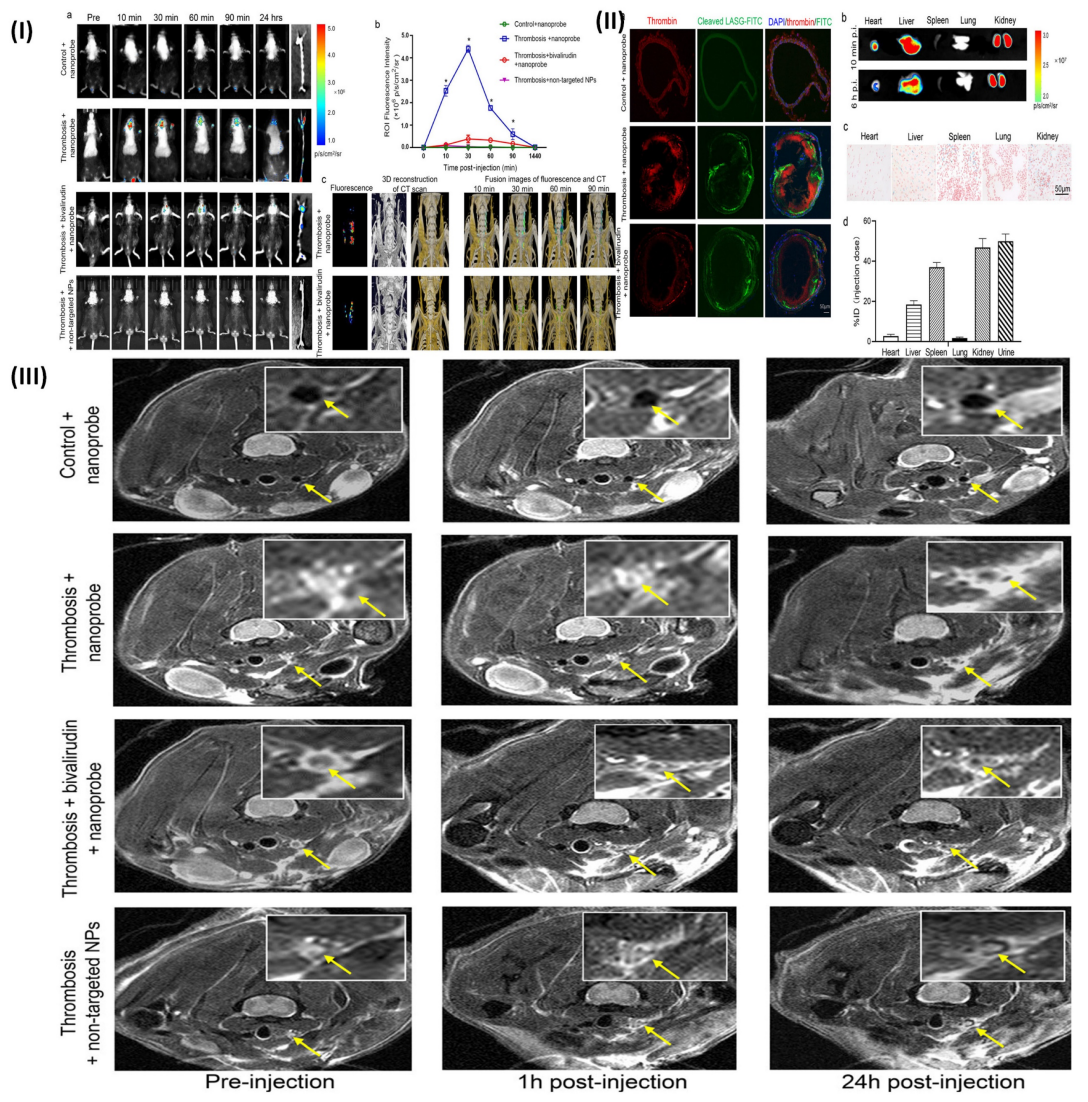
(I) (a) Fluorescence photos taken *in vivo* showing the thrombin activity before and after intravenous delivery of the non-targeted NPs or FITC-LASG-PEGylated Fe3O4 nanoprobe. Imaging of abdominal aorta and carotid artery *in vivo* using fluorescence (top row). (b) After the injection of either a non-targeted or FITC-LASG-PEGylated Fe_3_O_4_ nanoprobe, dynamic variations in fluorescence signal intensity were quantified. (c) Fluorescence and micro-CT scan pictures that have been combined (FITC-non-targeted peptide PEGylated Fe_3_O_4_ NPs as the non-targeted nanoprobe); (II) (a) Confocal microscopy images of carotid artery segment after injecting FITC-LASG- PEGylated-Fe3O4 nanoprobe. (b) Fluorescence imaging of extracted organs ex vivo 10 min and 6hour post injection (c) Prussian blue staining 1hour post-injection of excised organs. (d) ICP-MS analysis of urine samples collected post-injection and slices of excised organ tissue; (III) T2-weighted MRI of the carotid artery of mice undergoing thrombosis before to, immediately after, and 1 and 24 hours after nanoprobe or non-targeted NP administration. The location of arterial thrombi is shown by yellow arrows. Reproduced with permission from ref. 80. Fig. 5, Fig. 6, and Fig. 7 (ACS Publication).

However, Wen *et al.* developed dual-modality optical-MR theranostics NPs-based probes for imaging thrombi *in vivo*, with thrombosis targeting ability. Tobacco mosaic virus (TMV), an elongated rod-shaped plant virus, served as the basis for the nanoparticle. It was bioengineered and included the fibrin-binding peptides CREKA and GPRPP. These particles demonstrated selective fibrin binding *in vitro* in the presence of specific peptides using magnetic resonance (MR) and optical imaging. To convey contrast agents for dual-modality MR and optical imaging, these particles were loaded 77. Further Kwon *et al.* prepared a nanoprobe having diagnostic and therapeutic benefits using fluorescence/micro-CT dual imaging thrombus imaging in thrombosis are possible. To directly image thrombus using dual NIRF (near-infrared fluorescence) and micro-CT (micro-computed tomography) imaging, their team created a thrombin activatable fluorescent peptide (TAP) that included silica-coated gold NPs (TAP-SiO2@AuNPs). TAP molecules were employed as specific thrombin-activatable peptide thrombin probes 78. Nguyen *et al.* developed ultra-small zinc oxide NPs (ZnO-4/NPs) doped with iron ions and coated with a low molecular weight fucoidan. To balance magnetic and optical characteristics, the NPs (NP-0.50) were coated with a low molecular weight fucoidan. NPs with an iron/zinc ratio of 0.50 were used for further research work. Fucoidan-coated NP-0.50 are non-cytotoxic at doses up to 0.1 mg/mL for 24-48 hours, making them the best suitable contrast materials for fluorescence microscopy and MRI bimodal imaging 79. Wang et al, synthesized a new FITC-LASG-PEGylated Fe_3_O_4_ nanoprobe that responds to thrombin and can predict thrombin activity. They measure the thrombin activity via the thrombin's peptide substrate is responsible for the transition from a quenched fluorescence state to an active one. After injecting FITC-LASG-PEGylated Fe_2_O_3_ nanoprobe intravenously the fluorescence signal was recorded before 10, 30, 60, and 90 minutes to demonstrate thrombin-specific fluorescence imaging *in vivo*. After 10 minutes, the thrombotic lesion showed clear fluorescence signals from the injected probe, and the signal gradually reduced after 60 minutes. This may be connected to the elimination of cleaved FITC molecules from the bloodstream (as seen in Fig. 6Ia). Intense fluorescence signals observed in the LCCA following the mouse thrombus models indicate that thrombin was likely synthesized and greatly aggregated at the affected area of the LCCA, where it could then exert its procoagulant function. The visual signal strength was greater in superficial carotid arteries than in other types of vessels, and the RCCA showed a smaller fluorescence signal because there was little activated thrombin or cleaved FITC-peptide circulating in the systemic arteries. The background noise present in fluorescence images may also be to blame. By injecting the FITC-LASG-PEGylated Fe_2_O_3_ nanoprobe and the direct administration of the thrombin inhibitor bivalirudin into the tail veins of mice at the same time, they were able to further demonstrate the thrombin-responsive characteristic of the aforementioned nanoprobe. At each time point after probe insertion, the thrombotic lesion fluorescence signal was substantially attenuated compared to the thrombosis + nanoprobe group. To verify that thrombin was the only protease activating the nanoprobe *in vivo*, they conjugated a FITC peptide (non-substrate) to PEGylated Fe3O4 NPs and injected them into a mouse thrombus model via the tail vein. Fig. 6Ia, b demonstrated that the FITC-LASG-PEGylated Fe3O4 nanoprobe provided a more robust fluorescence signals in a mice thrombotic model compared to the negative control. The LCCA of the thrombosis along with the nanoprobe group had a much larger fluorescence signal compared to the other groups, as seen by ex vivo fluorescence images of detached carotid artery and tissue of the abdominal aorta. After 24 hours, there was essentially no fluorescence signal at the same place in all groups when the nanoprobe was re-injected (Fig. 6Ia, b). The FITC-LASG-PEGylated Fe3O4 nanoprobe may be used to distinguish between juvenile thrombi and existing clots in addition to evaluating thrombosis risks and the effectiveness of anticoagulant therapy (such as bivalirudin). Utilizing innovative CT contrast agents, micro-CT has allowed for significant progress in the anatomical monitoring of morphological alterations in small luminal structures. To assess LCCA blood flow, researchers here used contrast-enhanced micro-CT imaging with an ExiTron nano 6000 agent 10 minutes after injecting a FITCLASG-PEGylated Fe3O_4_ nanoprobe. ExiTron nano 6000 agents can produce equitable contrast and a strong signal augmentation of blood flow, as seen in the 3D reconstruction of micro-CT images. The FeCl_3_ wounded location of the LCCA showed remarkable filling signals of blood flow, in contrast to the RCCA, which showed no such signs. Pretreatment with bivalirudin can improve LCCA blood flow and reduce filling defects. Filling deficiencies in the LCCA were preferably clustered with the fluorescence signal created by the FITC-LASG-PEGylated Fe_3_O_4_ nanoprobe, as shown in 3D fusion images of micro-CT and fluorescence (Fig. 6Ic). Dissected LCCA tissues were stained with an antibody against thrombin to reveal the lesion's thrombin activity. Fig. 6IIa demonstrated that the separated FITC-LASG fragment's co-localized green fluorescence signal was simple to find in the thrombus lesion of the thrombosis + nanoprobe group. They employed a 7.0T MRI to look more closely at the thrombus in the mouse thrombosis model. In comparison with the control group and the bivalirudin therapy group, T1-weighted MRI in the thrombosis group showed a hyper-intense signal in the left carotid artery cavity. The T2-weighted MRI picture of the carotid lumen (Fig. 6III) likewise exhibited a comparable signal shift in the new and organized thrombus. Ex vivo fluorescence imaging of resected major organs was used to examine the biodistribution and clearance of the FITC-LASG PEGylated Fe_3_O_4_ nanoprobe injected intravenously at either 10 minutes or 6 hours post-injection. Fig. 6IIb shows that the separated fragments of FITC-LASG were largely accumulated in the kidney and liver after ex vivo imaging of several major organs, including the heart, liver, and kidney. The *in vivo* signal strength decreases drastically in deep tissue or organs like the kidneys, liver, and spleen; as a result, these structures are not displayed fluorescence in Fig. 6Ia. This is because fluorescence imaging is affected by animal hair, tissue depth, and skin color. In addition, the thrombi in the superficial carotid arteries were seen with higher fluorescence intensity by the dissociated FITC-LASG fragment than in the deeper organs. The *in vivo* biocompatibility of the FITC-LASG-PEGylated Fe3O4 nanoprobe was evaluated through histopathological examination of key organs. After injecting a FITC-LASG-PEGylated Fe3O4 nanoprobe, hematoxylin and eosin staining revealed no evidence of histopathological anomalies, such as tissue injury or inflammation. These results point to the FITC-LASG-PEGylated Fe_3_O_4_ nanoprobe's high biocompatibility and minimal cytotoxicity. The majority of the nanoprobe, as detected by Prussian blue staining 1 hour post-injection, is found in the kidneys, spleen, and liver. Signal strength varies throughout the spleen due to the various cleaved nanoprobe pieces. Thrombin cleaved the FITC-LASG-PEGylated Fe_3_O_4_ nanoprobe into the FITC-peptide fragment 1 and the peptide fragment 2-PEGylated Fe_3_O_4_ by enzymatic hydrolysis. Fig. 6IIb demonstrates that the liver and kidneys played a vital role in the accumulation and metabolism of cleaved FITC-peptide fragments. The spleen barely registered on the fluorescence spectrum. Prussian blue staining and inductively coupled plasma mass spectrometry (ICP-MS) analysis both demonstrated that 2-PEGylated Fe_3_O_4_ peptide fragments were predominantly localized in organs like kidneys, spleen, and liver (Fig. 6IIc). PEGylated Fe_3_O_4_ NPs can be ingested by macrophages in the spleen, they are present where most of the iron from an *in vivo* overload ends up being stored. Fig. 6IId shows that the spleen had a high signal intensity, in contrast to Fig. 6IIb. ICP-MS examination of blood and urine samples collected after intravenous (i.v.) administration of the nanoprobe to mice showed that the maximum plasma concentration (C_max_) and time to C_max_ (t_max_) were 0.5 hours and 68.4 3.8 pg/mL, respectively, and that the area-under-the-curve (AUC0) reached 260.6 pgxh/mL. Over 90% of the injection dosage (ID) of the nanoprobe had aggregated in the kidney, spleen, and liver after an hour, and by six hours after injection, around 50% of the ID had been removed in the urine (Fig. 6IId) 80. Recently Zhong *et al.* formulated a fibrin-specific nanoprobe for detecting thrombus composition by using PA and MRI. PLGA NPs surface (poly (lactic-co-glycolic acid) NPs) was altered by using CREKA peptide (fibrin-targeting peptide). For the direct detection of thrombi *in vivo*, formulated nanoprobe can act as a very sensitive sensor by merging Fe_3_O_4_ and NIR fluorochrome (IR780) 81.

## 5. Challenges in thrombus imaging and therapy

In day-to-day practice, pulmonary embolism (PE) remains a diagnostic challenge. There are undesirable consequences of both missed and excess diagnosis. In daily practice some aspects of PE diagnosis are still challenging and might take advantage of advances in imaging. It could be useful to provide an objective and quick assessment of degree of vascular blockage, whose association with the risk of PE recurrence is under controversy. Sub segmental PE's clinical applicability is still up for debate. The major concern of VTE (venous thromboembolism) diagnosis is *in vivo* characterization of clot form, for example for the distinction between tumoral and thrombus clot, or between a residual thrombus or an acute thrombus 99. Activated coagulation factors, fibrin, last by-product of coagulation cascade, and diverse epitopes on activated platelets are only a few of the distinct and nonexistent epitopes that are exposed by thrombi. Targeting agents for coagulation factors, such as peptides that identify activated coagulation factors specifically and antibodies that can consistently distinguish between the activated coagulation factors and circulating zymogens (proenzymes), are currently lacking. This has restricted the use of coagulation factors as molecular imaging targets 100. Contrast compounds for all biomedical theranostics modalities have particular advantages and disadvantages during early molecular theranostics. After years of research, fibrin and platelets continue to be the principal thrombus targets. All probes can easily image an acute thrombus, however targeting an older thrombus is still challenging.

Similar to anti-coagulation, usage of this medication is clinically required when pulmonary embolism, DVT, or coronary burst plaques are suspected despite the fact that it continues to interact with and frequently negates the usefulness of thrombus theranostics probes. The deficiency of clinical thrombus theranostics contrast agents indicates how difficult it will be to address these basic issues, which will necessitate an unconventional approach to design 101. The spatial resolution produced by the imaging hardware sets a detection threshold for conventional clinical imaging contrast agents 102. The contrast agents for medical imaging used majorly now are tiny compounds with a quick metabolism, a non-specific distribution, and possible toxicities 103 104. The clinical value is still restricted by the low penetration depth and the auto-fluorescence and scattering characteristics in different tissues 105. Low sensitivity for identifying abnormalities might also be caused by the target lesion's restricted fluorescence, possible blink and photobleaching effects, and other factors. Cost, extended imaging periods, motion artefact, and possible foreign body/implant artefacts are MRI's drawbacks. MRI contrast agents help in lesion identification and distinction from healthy tissues. Iodine is a primary element of intravenous CT contrast agents. The iodinated contrast agents have some drawbacks such as fast clearance, possible renal toxicity, generic blood pool distribution, and recorded adverse events/anaphylaxis. The several contrast agents which are nanosized are developed to get over these constraints and broaden the use of CT imaging 106. Radioactive exposure and expensive expense are two drawbacks of PET/SPECT 107. To introduce multimodality imaging into clinical practice, which is a relatively new modality, specific equipment with several imaging modules has to be created. These constraints include numerous and distinct designs that make it difficult to establish a standard criterion 108. Therapeutic use for a biodegradable or fast excreted nanoparticle imaging probe with low toxicity and a strong imaging signal. Several challenges affect various NPs that prevent their development into medicinal applications. The clearance is slow and more systemic circulation in comparison to imaging agents which are conventional, there may be an issue with increased systemic exposure to hazardous components of nanomaterial-based imaging agents 109 110. The NPs can create micron-scale aggregates that can disrupt microcirculation, especially in the capillary beds of the lungs, and cause inflammation and granuloma formation when injected into the bloodstream 111. It's crucial to look at how intravenous NPs tend to aggregate in plasma, especially when drugs are administered at high particle concentrations, and to assess the lung as a possible target organ 112.

Hence, one major difficulty in the field arises when a precise method is to be developed for determining the chemical identity of molecules by detecting their spatial placement on nanoscale curved surfaces 113. For biological applications, NPs may be too hydrophobic, or there may be issues with the ligands' toxicity. So, the field's goal is to quantify the percentage of designer-coated NPs that bind to their intended targets and to comprehend the reasons why this event fails 114. In order to be used for cellular assay or imaging, there must be no interference of evaluated NPs with typical processes of cells including signal transduction, receptor trafficking, or functions of the membrane to be used for cellular assay or imaging. The techniques used now to detect physiologically relevant targets are labor-intensive, challenging to conduct, and only partially multiplexable. There are still having significant problems using the power of NPs for theranostic approach with hypersensitivity due to non-specific binding and NPs aggregation 115.

## 6. Future perspective

The future seems promising for synthesized and engineered theranostics probes, like peptide probes and nanoparticle probes in biomedical theranostics. The development of imaging probes that are non-toxic, biocompatible, biodegradable and circumvent non-specific organ uptake and RES should continue. Researchers in this field should be aware of the difficulty in collecting and analyzing massive data sets acquired by employing nanoparticle contrast agents as well as any potential expenses associated with adopting advanced imaging equipment. In thrombosis's management, molecular imaging in clinical trials has the potential to play a vital role, a condition for which diagnostic imaging is currently widely employed 116. Integrating targeting moiety, external stimulating forces like magnetic fields and ultrasound, shear-activated therapeutics, and the addition of imaging/diagnostic agents are some of the multifunctional strategies used to create advanced targeted antithrombotic nanomedicines, which will likely enable the creation of a variety of novel products for effective antithrombotic therapy. To increase the specificity and efficacy, theranostic nanomedicines which include therapeutic and diagnostic components that can be used as a potential delivery mechanism for individualized antithrombotic therapy (tailored for each patient that has thrombosis) 117. Targeting methodologies and the use of nano-delivery systems for co-delivering thrombolytic medications provide a bright future for prolonging drug therapy in a shorter amount of time and significantly increasing therapeutic efficacy through synergistic effects 118 119. Overall, individuals with thrombotic illness may greatly benefits from the therapeutic use of molecular theranostics. Moreover, the regulatory complexity of these sorts of combination approaches should be carefully taken into account when developing new concepts including NPs with various functionalities (such as imaging and therapy) 120.

## Conclusion

Several methods for employing NPs and various probes that emerged to satisfy the diagnostic needs of contemporary molecular medicine as a result of the fast growth of nanotechnology over the past years. NPs have been incredibly useful in clinical medicine, particularly in targeted drug delivery and diagnosis via imaging. Molecular imaging may identify thrombotic disorders and plaques which are susceptible to high-risk, by choosing the appropriate biomarkers/targeting epitopes and contrast agents. It will be possible to develop more personalized and targeted treatments by having a better understanding of how the disease is progressing, with the significant use of molecular imaging. The many molecular markers that are expressed at distinct illness stages make it easier to assess the effectiveness of therapy. There are different types of probes which include peptides and NPs that can be used for thrombosis imaging, including iron oxide NPs and gold NPs. We can significantly enhance both conventional biological imaging of cells and tissues and contemporary imaging techniques for a variety of body locations.

Furthermore, NPs can be employed effectively not just for imaging purposes but also for targeted drug delivery in the treatment of thrombosis. NPs can be formulated to specifically target the blood clot, delivering drugs straight to the site of the thrombosis and reducing the risk of the side effects from systemic drug delivery. Overall, employing NPs for thrombosis imaging and treatment holds promise for improving diagnosis and therapy for this potentially life-threatening condition.

## Figures and Tables

**Table 1:** Several theranostic nanomedicine for thrombosis

S.No.	Type of Particle	Imaging technique	Imaging Moiety/Probe	Targeting site	Reference
1.	Trilysine-protected Gd^3+^-DTPA complex	MRI	Gd-DTPA	Fibrin	56
2.	Bi-α AP-CA (specific bimodal α -antiplasmin- based contrast agent)	MRI	Gadolinium-diethylene triamine pentaacetic acid (Gd-DTPA), Rhodamine	α -antiplasmin	57
3.	USPIO-FUCO (ultra-small superparamagnetic iron oxide nanoparticles with fucoidan)	MRI		Activated platelets	58
4.	DCIONs (dual-contrast iron oxide nanoparticles) magnetic	MRI-7 T	-	Activated platelets	59
5.	CLIOs	MRI		Fibrin	60
6.	IONCs- Gd-DTPA (iron oxide nanoclusters with Gd-DTPA)		Gd-DTPA	Fibrin	61
7.	TargPFCs	^19^F MRI		activated platelets	62
8.	Fucoidan-MBs (Fucoidan-microbubble)	US		P-selectin	63
9.	Hyaluronic acid (HA) polymer-phospholipid shell- MBs	US		Thrombus	64
10.	AuNPs-PEG (gold nanocrystals)	CT		Thrombus	65
11.	GC-AuNPs (glycol-chitosan-coated gold nanoparticles)	CT		Fibrin and t-PA	66
12.	FA-Fe_3_O_4_-AuNPs (aptamer functionalized superparamagnetic gold-coated iron-oxide nanoparticles)	CT		Thrombus	67
13.	Mesoporous silica	NIR		Platelets	68
14.	TTQ-PEG-c(RGD)	NIR	TTQ	Active platelet GPIIb/IIIa	69
15.	TIRO-CyAl5.5 (polyethylene glycol (PEG)- tirofiban analogues)		Cy7	Platelets	70
16.	lanthanide-doped scintillator nanocrystals (NCs)	NIR-XEL		Thrombus	
17.	Phthalocyanine-based clot homing probe	NIR		Thrombus	71
18.	GNRs (gold nanorods)	PAI		ICAM-1, and E-selectin	72
19.	cRGD-PDI NPs	PAI			73
20.	fibrin-specific peptide	MRI/PET/	Fluorophore (OI) or Gd or ^64^Cu	Fibrin	74
21.	peptide-chelate conjugates	MRI/PET	64Cu-DOTA	Fibrin	75
22.	SPION (superparamagnetic iron-oxide nanoparticle)	NIR/MRI	IR783	Microthrombus	76
23.	Tobacco mosaic virus NP (CREKA and GPRPP fibrin-binding peptides)	Optical/MRI		Thrombus	77
24.	TAP-SiO2@AuNPs	NIRF/ micro-CT	Cy5.5	Thrombus	78
25.	ZnO-4/NPs	MRI/ Fluorescence		Thrombus	79
26.	FITC-LASG-PEGylated Fe_3_O_4_	Fluorescence/ micro-CT/MRI		Thrombus	80
27.	PLGA NPs	PAI/MRI/NRI	IR780	Fibrin	81

## Abbreviations

NPs: Nanoparticles
NETs: Neutrophils
IL-8: Interleukin-8
DVT: Deep vein thrombosis
GP Ib: Glycoprotein Ib
GP IIb/IIIa: Glycoprotein IIb/IIIa
vWF: von Willebrand factor
tPA: *Tissue plasminogen activator*
PAI-1: P*lasminogen Activator Inhibitor* 1
RBCs: Red blood cells
WBCs: White blood cells
TF: Tissue Factor
MI: *Myocardial Infarction*
AIS: *Arterial ischemic stroke*
64Cu-FBP8: 64Cu-Fibrin Binding Probe-8
PFPE: Perfluoropolyether
CLIOs: Cross-linked iron oxide *nanoparticles*
PBN's: Peptide-based nanoprobes
TargPFCs: Targated perfluorocarbons
MBs: Microbubbles
CPS: Contrast pulse sequencing
HA-MBs: Hyaluronic acid-microbubbles
NIR-XEL: Near-infrared X-ray-excited luminescence
ICAM-1: *Intercellular Adhesion Molecule 1*
PBS: Phosphate-buffered solution
LCCA: Left common carotid artery
RCCA: Right common carotid artery
DCIONs: Dual-contrast iron oxide nanoparticles
Fib-GC-AuNPs: Fibrin-targeting peptides glycol-chitosan-coated gold nanoparticles
Fe_3_O_4_-AuNPs: Superparamagnetic gold-coated iron-oxide nanoparticles
PASK: Proline, alanine, serine, and lysine
MS@PASKE: Mesoporous silica- Proline, alanine, serine, and lysine
cRGD-PDI NPs: cRGD-perylene-3,4,9,10-tetracarboxylic diimide nanoparticles
TAP-SiO2@AuNPs: Thrombin activatable peptide-silica-coated gold nanoparticles
THP-1: Human monocytic cells
BCN: Bicyclo[6.1.0]nonyne
